# Morphological and Phylogenetic Appraisal of Novel and Extant Taxa of Stictidaceae from Northern Thailand

**DOI:** 10.3390/jof7100880

**Published:** 2021-10-19

**Authors:** De-Ping Wei, Dhanushka N. Wanasinghe, Eleni Gentekaki, Vinodhini Thiyagaraja, Saisamorn Lumyong, Kevin D. Hyde

**Affiliations:** 1 CAS Key Laboratory for Plant Diversity and Biogeography of East Asia, Kunming Institute of Botany, Chinese Academy of Sciences, Kunming 650201, China; wei_deping@cmu.ac.th (D.-P.W.); dnadeeshan@gmail.com (D.N.W.); 2 Center of Excellence in Fungal Research, Mae Fah Luang University, Chiang Rai 57100, Thailand; gentekaki.ele@mfu.ac.th (E.G.); vino.thiyagaraja@gmail.com (V.T.); 3 Department of Entomology and Plant Pathology, Faculty of Agriculture, Chiang Mai University, Chiang Mai 50200, Thailand; 4 Mushroom Research Foundation, 128 M.3 Ban Pa Deng T. Pa Pae, A. Mae Taeng, Chiang Mai 50150, Thailand; 5 World Agroforestry Centre, East and Central Asia, Kunming 650201, China; 6 Centre for Mountain Futures, Kunming Institute of Botany, Kunming 650201, China; 7 School of Science, Mae Fah Luang University, Chiang Rai 57100, Thailand; 8 Research Center of Microbial Diversity and Sustainable Utilization, Chiang Mai University, Chiang Mai 50200, Thailand; scboi009@chiangmai.ac.th; 9 Department of Biology, Faculty of Science, CHiang Mai University, Chiang Mai 50200, Thailand; 10 Academy of Science, The Royal Society of Thailand, Bangkok 10300, Thailand; 11 Innovative Institute of Plant Health, Zhongkai University of Agriculture and Engineering, Haizhu District, Guangzhou 510225, China

**Keywords:** lichenization, new species, non-lichenized fungi, Ostropales, phylogeny, taxonomy

## Abstract

Stictidaceae comprises taxa with diverse lifestyles. Many species in this family are drought resistant and important for studying fungal adaptation and evolution. Stictidaceae comprises 32 genera, but many of them have been neglected for decades due to the lack of field collections and molecular data. In this study, we introduce a new species *Fitzroyomyces hyaloseptisporus* and a new combination *Fitzroyomyces* *pandanicola*. We also provide additional morphological and molecular data for *Ostropomyces pruinosellus* and *O. thailandicus* based on new collections isolated from an unidentified woody dicotyledonous host in Chiang Rai, Thailand. Taxonomic conclusions are made with the aid of morphological evidence and phylogenetic analysis of combined LSU, ITS and mtSSU sequence data. Characteristics such as the shape and septation of ascospores and conidia as well as lifestyles among genera of Stictidaceae are discussed.

## 1. Introduction

Nannfeldt [1] established Ostropales to accommodate Ostropaceae, which was later synonymized under Stictidaceae [2]. The taxonomic position of Ostropales is within Ostropomycetidae (Lecanoromycetes and Ascomycota), which was assigned based on multigene phylogenetic analyses [3]. The outlines of Ostropales have been revised in several studies, and the number of families in the order has been subject to multiple changes over time [4,5,6,7]. Previously, 14 families had been included in Ostropales, namely, Coenogoniaceae, Gomphillaceae (syn. Solorinellaceae), Graphidaceae, Gyalectaceae, Odontotremataceae, Phaneromycetaceae, Phlyctidaceae, Trichotheliaceae (syn. Myeloconidaceae and Porinaceae), Protothelenellaceae (syn. Thrombiaceae), Sagiolechiaceae, Spirographaceae, Stictidaceae, Thelenellaceae and Thelotremataceae [2,4,5,6,7,8,9]. However, multigene phylogenies have ultimately resulted in the transfer of most families into different orders, such as Baeomycetales, Thellenellales, Graphidales and Gyalectales. Presently, Stictidaceae is the only family assigned to Ostropales [10].

**Figure 1:**
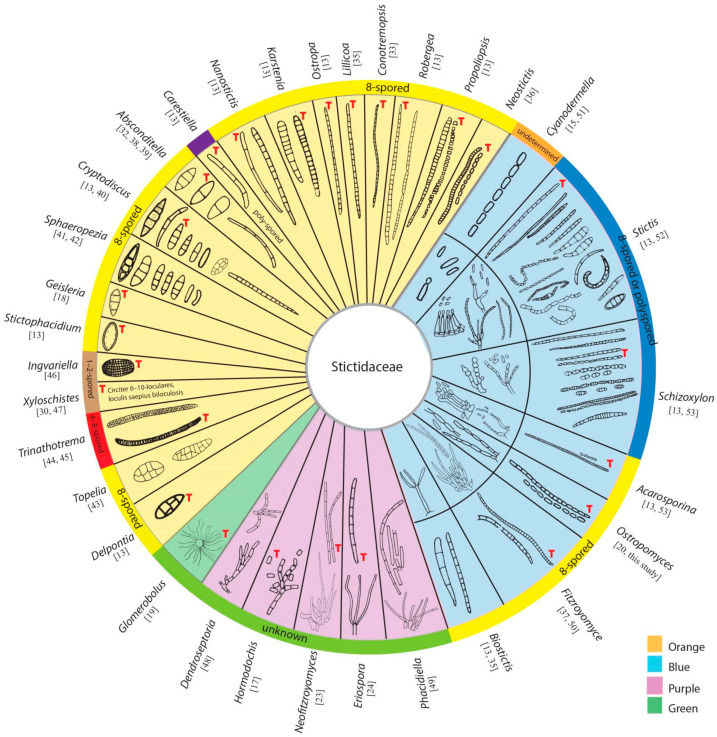
Morphology of ascospores, conidia and conidiogenesis of 32 genera in Stictidaceae. The orange area contains genera known only from sexual morphs; the area in purple comprises genera that were established based only on asexual morphs; and the area in blue encompasses genera that were described from both sexual and asexual morphs, while the area in green consists of a single genus, which reproduces via sticky propagules rather than sexual or asexual spores. The number of ascospores per ascus for each genus is noted in the outermost circle. The ascospores and conidia indicated with the red letters “T” were redrawn from the type species. The original references of these characters are cited for each genus.

Stictidaceae was introduced based on *Stictis* Pers, a saprobic genus, which is characterized by apothecioid ascomata, cylindrical asci and filiform ascospores [11,12]. Subsequently, researchers extended the membership of this family to include both morphologically and phylogenetically related genera [13,14,15,16,17,18,19]. The generic composition of Stictidaceae has been outlined by listing the genera name, but it lacks detailed annotations [4,5,6,7]. Thiyagaraja et al. [20] introduced the novel genus *Ostropomyces* and accepted 32 genera following the previous outline. Molecular data are lacking for *Biostictis*, *Conotremopsis*, *Delpontia*, *Dendroseptoria*, *Karstenia*, *Lillicoa*, *Nanostictis*, *Propoliopsis*, *Stictophacidium* and *Topelia.* The remaining 22 genera, including *Absconditella*, *Acarosporina*, *Carestiella*, *Cryptodiscus*, *Cyanodermella*, *Eriospora*, *Fitzroyomyces*, *Geisleria*, *Glomerobolus*, *Hormodochis*, *Ingvariella*, *Neofitzroyomyces*, *Neostictis*, *Ostropa*, *Ostropomyces*, *Phacidiella*, *Robergea*, *Schizoxylon*, *Sphaeropezia*, *Stictis*, *Trinathotrema* and *Xyloschistes*, have been confirmed as members of Stictidaceae by phylogenetic methods. *Thelopsis* historically has been assigned to Stictidaceae, however, recent phylogenetic analyses of combined LSU, mtSSU and RPB2 sequences have placed this genus outside this family, with the type species placing within *Gyalecta* (Gyalectaceae) [21]. Stictidaceae currently comprises 32 genera, including 19 sexual (in orange area, Figure 1), five asexual genera (in purple area) and seven genera for which both morphs have been observed (in blue area). Uniquely within Stictidaceae, *Glomerobolus* (in green area), a monotypic genus, reproduces via discharging a sticky propagule on fresh substrates rather than by producing sexual or asexual spores [19,22]. Molecular analyses have contributed substantially towards elucidating the natural classification of Stictidaceae [2,20]. Studies have attempted to ascertain phylogenetic relationships within and among species of Stictidaceae using different sequence datasets, such as individual SSU [12] and LSU [15,16,17,23,24] genes, as well as LSU-mtSSU-RPB2 [2] and LSU-ITS-mtSSU combined matrices [25,26,27,28,29]. However, inferring robust intergeneric relationships has been challenging given the limited sampling of Stictidaceae species.

Sexual morphs have been described for 26 genera in Stictidaceae. Members of this family typically develop apothecioid ascomata, except for *Geisleria* and *Ostropomyces*, where both possess perithecioid ascomata [18,20]. Most genera of Stictidaceae have cylindrical to clavate 8-spored asci. Nonetheless, the number of ascospores per ascus differs among genera (Figure 1). For example, *Xyloschistes* and *Ingvariella* are 1–2-spored [30], and *Trinathotrema* is 4–8-spored [31], while *Carestiella*, *Schizoxylon* and *Stictis* contain poly-spored species [13]. Deviation from 8-spored asci could have resulted from the abortion or disarticulation of primary ascospores [13]. Significant variation also exists among Stictidaceae genera regarding ascospore characteristics that might be informative in generic delimitation. The elongated clavate, long-cylindrical to filiform and multiseptate (>3-septate) ascospores can be observed in *Absconditella* [32], *Cryptodiscus* [13,33], *Conotremopsis* [34], *Karstenia* [13], *Lillicoa* [35], *Nanostictis* [13], *Ostropa* [13], *Robergea* [13], *Acarosporina* [13], *Biostictis* [35], *Cyanodermella* [15], *Fitzroyomyce* [36,37], *Neostictis* [36], *Ostropomyces* [20], *Propoliopsis* [13], *Schizoxylon* [13] and *Stictis* [13] (Figure 1). The latter nine genera contain species whose ascospores can disarticulate into simple to septate part-spores or irregular fragments. The fusiform, oblong, ellipsoidal to short clavate and few-celled (0–3-septate) ascospores are observed in *Absconditella* [38,39], *Biostictis* [35], *Cryptodiscus* [40], *Geisleria* [18], *Sphaeropezia* [41,42], *Stictis* [13] and *Stictophacidium* [13]. The oblong, ellipsoidal, obovoid to cylindrical and submuriform ascospores are present in *Delpontia* [13], *Topelia* [43] and *Trinathotrema* [44,45]. The oblong and muriform ascospores have been noted in *Ingvariella* [46] and *Xyloschistes* [30,47].

Asexual morphs have been observed in 12 genera within Stictidaceae. In most cases, their conidiomata are pycnidial, with the exception of *Biostictis* (with hyphomycetous sporulation structures) [35] and *Dendroseptoria* (with sporodochial fruiting bodies) [48]. Four modes of conidiogenesis have been observed in this family, including blastic-phialidic (i.e., *Dendroseptoria* [48], *Neofitzroyomyces* [23], *Phacidiella* [23,49], *Fitzroyomyce* [50], *Cyanodermella* [51] and *Stictis* [52]), blastic-sympodial (i.e., *Eriospora* [24], *Biostictis* [35] and *Acarosporina* [53]), blastic-retrogressive (i.e., *Ostropomyces* [20] and *Schizoxylon* [53]) and thallic-arthric (i.e., *Hormodochis* [17] and *Schizoxylon* [53]). Stictidaceae formed aseptate, one-celled to multiseptate, ellipsoidal to long cylindrical and occasionally tetraradiate conidia (see Figure 1).

Stictidaceae species commonly occur on bark, leaves, stems and wood of various plant hosts in terrestrial habitats [25] and have broad geographic distribution in Africa, Asia, Europe and North America [13]. This family contains a broad diversity of lifestyles, ranging from saprobes, pathogens and endophytes to lichens. Lichenicolous species have been recorded in *Cryptodiscus*, *Nanostictis* and *Sphaeropezia* [33,42,54], while *Schizoxylon albescens*, *Stictis confusa* and *Stictis populorum* show optionally lichenized lifestyles depending on the associated substrates [55,56]. Thus, Stictidaceae played an important role in understanding how fungi can adapt to various successional substrates during habitat succession by switching lifestyle [55].

In this study, we aim to clarify the taxonomic placement of new species (*Fitzroyomyces hyaloseptisporus* sp. nov. and *Fitzroyomyces pandanicola* comb. nov) and two previously described species (*Ostropomyces pruinosellus* and *O. thailandicus*) by using morphological and multigene-based phylogenetic analyses. To provide an identification scheme for all genera of Stictidaceae, the diversity of ascospore/conidia morphology and the mode of conidial development is summarized. Descriptions of sexual and asexual morphs of Stictidaceae are refined to keep abreast of current literature. The lifestyles of genera in Stictidaceae are mapped on the phylogenetic tree in order to visualize these features in a phylogenetic context and to explore the transition of nutrition modes in Stictidaceae.

## 2. Materials and Methods

### 2.1. Sample Collections and Isolation

Specimens were collected from an unidentified woody dicotyledonous plant in northern Thailand. Pure cultures were obtained via single spore isolation as outlined in Senanayake et al. [57]. The obtained cultures were deposited in Mae Fah Luang University culture collection (MFLUCC), Chiang Rai, Thailand, and herbarium specimens were deposited in Mae Fah Luang University Herbarium (Herb. MFLU). The Faces of Fungi numbers were obtained following Jayasiri et al. [58], and species names were registered in Index Fungorum (2021).

### 2.2. Morphological Studies

Specimens were examined with a Motic SMZ 168 stereomicroscope. Hand sections of the ascomata and conidiomata were mounted in water for microscopic studies and photomicrography. A Congo red solution was used to observe asci and paraphyses. The key structures such as ascomata, exciple, paraphyses, asci, ascospores, conidiogenous cells and conidia were observed by using a Nikon ECLIPSE 80i compound microscope, and they were photographed with a DS-Ri2 camera attached to the compound microscope. The measurements were taken with the Tarosoft (R) Image Frame Work program, while images used for figures were processed with Adobe Photoshop CS3 (Version 15.0.0, Adobe^®^, San Jose, CA, USA).Extended version 10.0 (Adobe Systems, San Jose, CA, USA).

### 2.3. DNA Extraction, PCR Amplification and Sequencing

Genomic DNA was extracted separately from both fresh fungal mycelia growing on potato dextrose agar (PDA) media and fruiting bodies using Biospin Fungus Genomic DNA Extraction Kit (BioFlux^®^, Hangzhou, China) following the protocol of the manufacturer. Polymerase chain reaction (PCR) was carried out for three partial gene fragments including large subunit ribosomal rRNA (LSU), internal transcribed spacers (ITS) and mitochondrial small subunit ribosomal rRNA (mtSSU) with primers LR0R/LR5 [59], ITS5/ITS4 [60] and mrSSU1/mrSSU3R [61], respectively. Amplification reactions were performed in 25 μL of PCR mixtures containing 8.5 μL of ddH2O, 12.5 μL 2×PCR Master Mix (Bioteke Corporation, Beijing, China), 2 μL of DNA template and 1 μL of each primer. PCR amplifications of LSU and ITS genes were performed as described by Wanasinghe et al. [33], while mtSSU gene was amplified following Zoller et al. [61]. Amplified PCR products were sequenced in Tsingke (Kunming, China). Sequences generated in this study were deposited in GenBank, and accession numbers were obtained (see Table 1).

### 2.4. Sequence Alignment and Phylogenetic Analyses

Raw sequences generated in this study were assembled with Sequencing Project Management (SeqMan) [62]. Megablast search using the newly generated sequences as queries was performed to check for contamination and to reveal closely related taxa in the GenBank nucleotide database. The available taxa representing genera in Stictidaceae are listed in Table 1. Each gene matrix was independently aligned with MAFFT (http://mafft.cbrc.jp/alignment/server/, accessed on 31 July 2021) [63]. Uninformative gaps and ambiguous regions were removed using Trimal available on the Phylemon 2.0 online platform [64]. Trimmed alignments were combined with Sequence Matrix v. 1.7.8 [65]. The combined alignment was used for maximum likelihood (ML) and Bayesian inference (BI) analyses.

Maximum likelihood analysis was performed using RAxML-HPC2 on XSEDE (8.2.10) in CIPRES Science Gateway V. 3.3 [66] by employing default parameters but with the following adjustments: Bootstrap iterations were set to 1000, and substitution model was set to GTR+GAMMA+I. The optimal nucleotide substitution models used for Bayesian analysis were independently selected for each locus under Akaike information criterion (AIC). Bayesian analysis was performed with MrBayes 3.2.7a in CIPRES Science Gateway v. 3.3 [67] in order to infer posterior probabilities (PP) [68,69] with Markov Chain Monte Carlo sampling (MCMC). Six simultaneous Markov chains were run for 2,000,000 generations, and trees were sampled every 1000 generations, resulting in 2000 trees. The first 25% of trees, representing the burn-in phase of the analyses, were discarded, while the remaining 75% of trees were used to calculate PP in the majority rule consensus tree.

Phylograms were visualized with FigTree v1.4.0 program [70] and edited in Microsoft power point (2016) and Adobe Illustrator^®^ CS3 (Version 15.0.0, Adobe^®^, San Jose, CA, USA). The final combined alignment was submitted to TreeBASE with submission number 28693 (http://purl.org/phylo/treebase/phylows/study/TB2:S28693, accessed on 31 July 2021).

## 3. Results

### 3.1. Phylogenetic Analysis

Maximum likelihood phylogenetic analysis was conducted using combined LSU, ITS and mtSSU sequence data of 70 representative taxa of Stictidaceae, two species of Thelenellaceae and three species of Trapeliaceae. The tree is artificially rooted to *Orceolina kerguelensis*, *Placopsis perrugosa* and *Trapelia placodioides* in Trapeliaceae, following Baloch et al. [7]. Of the 32 genera within Stictidaceae, molecular data are available for 22, 17 of which contain the type species. Thus, the sequences from all 22 genera are used in the phylogenetic analysis herein. Alignment comprised 2140 characters, including gaps (LSU: 841; ITS: 569; mtSSU: 730), of which 1001 characters were constant, 203 variable characters were parsimony-uninformative and 936 (43%) characters were parsimony-informative. The ML analysis of the combined dataset yielded a best scoring tree with a final ML optimization likelihood value of −26,748.800570. The alignment had 1332 distinct alignment patterns, with 30.23% completely undetermined characters and gaps.

**Figure 2:**
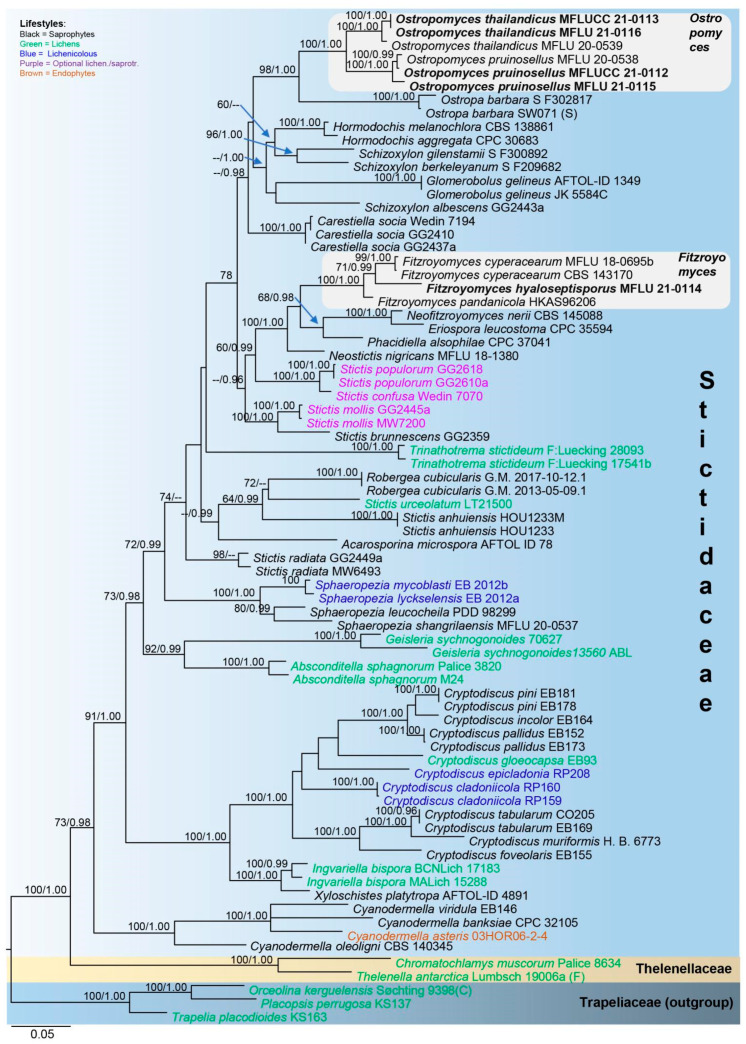
Phylogram generated from maximum likelihood analysis based on combined LSU, ITS and mtSSU sequence data. The tree was inferred from 75 taxa and 2140 sites. Maximum likelihood bootstrap support (MLBS) equal to or greater than 60% and Bayesian posterior probabilities (PP) equal to or higher than 0.95 are placed on the nodes in this order. The newly generated sequences are indicated in bold font.

In the phylogenetic tree (Figure 2), Cryptodiscus, Cyanodermella, Fitzroyomyces, Ostropomyces and Sphaeropezia are monophyletic, while Schizoxylon and Stictis are polyphyletic. Fitzroyomyces, Eriospora, Neofitzroyomyces and Phacidiella form a clade with high statistical support (100% MLBS/1.00 PP). The asexual morphs of these genera commonly occur on plant leaves, whereas the sexual morph of Fitzroyomyces has been found on dead woody stems [23,24,49,50]. The lichenized genera Geisleria and Absconditella formed sister clades with high support (92% MLBS/0.99 PP). Xyloschistes platytropa, a saprobe [30], is sister to Ingvariella bispora, a lichen [46], with maximum support (100% MLBS/1.00 PP). Our strain MFLUCC 21-0111 together with F. cyperacearum and F. pandanicola formed a monophyletic clade with maximum support (100% MLBS/1.00 PP). The new strains MFLUCC 21-0112 (from mycelia) and MFLU 21-0115 (from fruiting bodies) clustered with Ostropomyces pruinosellus with maximum statistical support values (100% MLBS/1.00 PP). The strains MFLUCC 21-0113 (from mycelia) and MFLU 21-0116 (from fruiting bodies) grouped with O. thailandicus also possessed maximum support (100% MLBS/1.00 PP).

### 3.2. Taxonomy

#### 3.2.1. Stictidaceae Fr. (as ‘Stictei’), Summa Veg. Scand., Sectio Post. (Stockholm): 345 (1849) Amend

Notes: Stictidaceae was briefly described as having apothecioid to perithecioid ascomata, unbranched paraphyses, cylindrical asci and filamentous ascospores [12,25,37]. Species in this family show high variation in ascospore shape and septation. Thus, a high diversity in characteristics emphasized the need for further revision of Stictidaceae. In this study, we amended Stictidaceae by inserting 1-spored to poly-spored asci, subglobose, oblong, fusiform, clavate, vermiform aseptate, few-celled and submuriform to muriform ascospores. We also summarize features of the asexual morphs of this family for the first time.

Saprotrophic, lichenized or optionally lichenized on the wood, bark, stem and leaves of various plant hosts, or are lichenicolous on other microfungi or living as parasites and endophytes of living plants. Sexual morph: *Ascomata* immersed or semi-immersed to superficial, perithecioid or apothecioid. They are gregarious, opening by the entire pore or transverse slit. *Discs* vary in color ranging from white, grey and brown to dark and are usually pruinose. *Exciple* typically consists of interwoven hyphae, sometimes contains crystalline inclusions. *Periphysoids* present or not. *Hymenium* comprising asci and paraphyses, commonly enclosed in a thick gelatinous matrix. The *subhymenium* is hyaline or pigmented and composed of angular cells. The *paraphyses* is filiform, simple or branched and sometimes apically enlarged, circinate and adhering to form an epithecium. *Asci* are cylindrical to clavate, 1-spored to poly-spored and usually possess thickened apex. *Ascospores* are typically hyaline, variable in shape and septation and span from subglobose, oblong, fusiform, clavate, vermiform, cylindrical to filiform and from aseptate, few-celled, multiseptate to muriform, breaking into part-spores or not. Asexual morph: *Conidiomata* are pycnidial or sporodochial and rarely hyphomycetous; they are globose to disc-shaped, hyaline, subhyaline, green and pale brown to dark. *Conidiophores* are hyaline to subhyaline, cylindrical, septate or aseptate and simple or branched. *Conidiogenous cells* are hyaline to subhyaline, subcylindrical to doliiform and usually simple, smooth-walled, enteroblastic, holoblastic or holothallic. *Conidia* are hyaline to dark olivaceous, doliiform, cylindrical, acicular, filiform, one-celled to multiseptated and occasionally staurosporous.

#### 3.2.2. *Fitzroyomyces* Crous, Persoonia 39: 389 (2017)

Index Fungorum number: IF 823395, three morphological species and three species with molecular data.

Type species—Fitzroyomyces cyperacearum Crous (2017).

Notes: *Fitzroyomyces* was described to accommodate *F. cyperacearum* (=*F. cyperi*), the asexual morph of which was recorded from the leaves of Cyperaceae in Australia [50]. The sexual morph of *F. cyperacearum* has been recorded from a dead stem of *Clematis subumbellata* and *Epilobium angustifolium* in Thailand [36,37]. *Fitzroyomyces* is characterized by immersed and cupulate apothecia with a crystalliferous exciple and cylindric-clavate asci with a thickened apex and cylindrical-filiform, multiseptated and hyaline ascospores with one to three deep constrictions, which seemingly contribute to the fragmentation.

**Figure 3:**
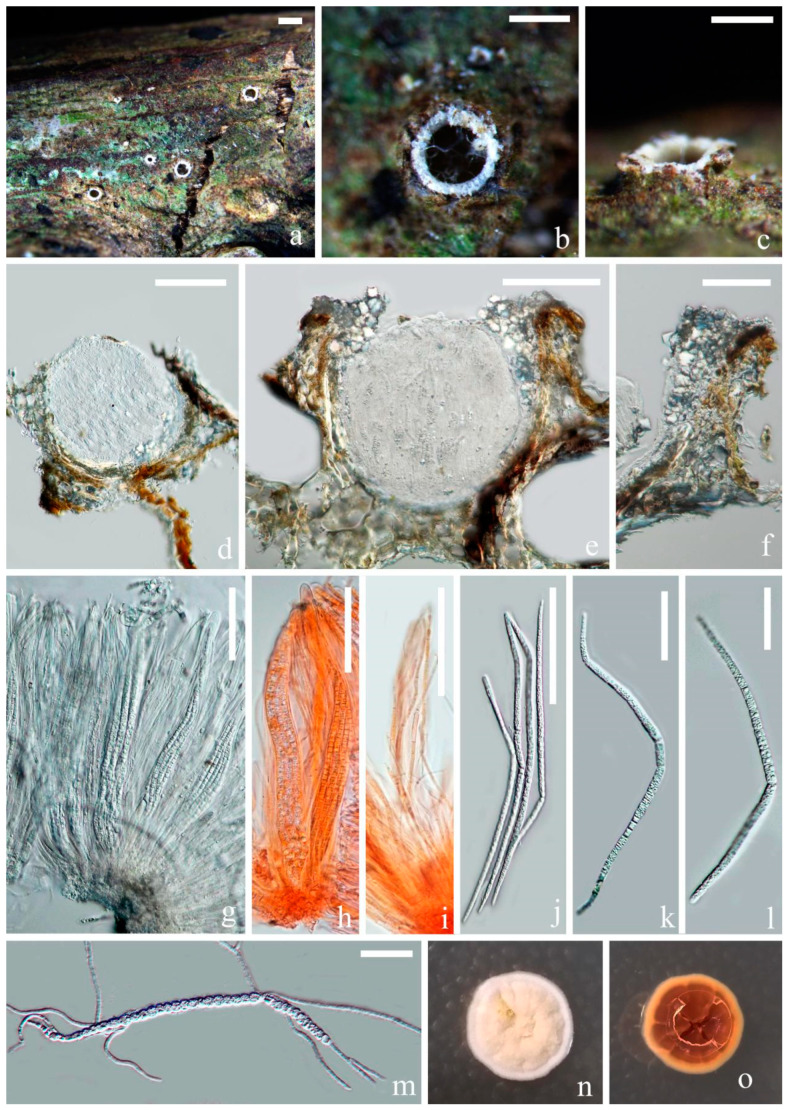
*Fitzroyomyces hyaloseptisporus* (MFLU 21-0114, holotype). (**a**–**c**) Apothecia on host; (**d**,**e**) vertical sections through apothecia; (**f**) excipulum; (**g**,**h**) asci; (**i**) paraphyses growing from subhymenium; (**j**–**l**) ascospores; (**m**) germinating ascospore; (**n**,**o**) upper and lower view of culture on PDA agar after incubation for 16 days. Scale bars: (**a**) = 500 μm; (**b**,**c**) = 200 μm; (**d**,**e**) = 100 μm; (**f**–**i**) = 50 μm; (**j**–**m**) = 30 μm. ((**h**,**i**)) mounted in Congo red solution.)

*Fitzroyomyces hyaloseptisporus* D. P. Wei and K. D. Hyde, sp. nov. Figure 3.

Index Fungorum number: IF558678; Facesoffungi number: FoF 10095.

Etymology: The specific epithet ‘‘hyaloseptisporus’’ refers to the fungus having hyaline and multi-septate ascospores.

Saprobic on dead stem of an unidentified climbing plant in a tropical forest. Sexual morph includes the following: *Apothecia*, 140–200 × 150–200 μm ($\bar{x}$ = 170 × 175, *n* = 5), subglobose, gregarious, immersed at first and opening by entire pore at maturity; *Disc* deeply cupulate, pale creamy and with white-pruinose margin. *Exciple*, 6–20 μm ($\bar{x}$ = 10, *n* = 25), comprising hyaline cells of textura angularis, thin, without periphysoidal layer and with crystalliferous upper part. *Hymenium* is embedded in a gelatinous matrix, comprising paraphyses and asci and can easily slip away from exciple when dry. The *Sub-hymenium* is thin, consisting of hyaline cells of textura angularis. *Epithecium* is absent. *Paraphyses* is 1–3 μm ($\bar{x}$ = 2, *n* = 25) in the wide, hyaline, filiform, unbranched and septate and apically cohering. *Asci* ranged at 165–200 × 10–25 μm ($\bar{x}$ = 180 × 15, *n* = 25), 8-spored, unitunicate, cylindric-clavate and broadest at middle part, with thickened and rounded apex. *Ascospores* are 150–200 × 3.5–6 μm ($\bar{x}$ = 172 × 5, *n* = 25), hyaline and long-cylindrical, with acute ends; they are frequently multiseptate, up to 58-septa and finely guttulate when mature. They have a spiral arrangement, commonly presenting 1–3 prominent constrictions, where the entire ascospores easily break into different size of fragments. *Cells of ascospores* are 2–7 μm ($\bar{x}$ = 4.5, *n* = 30) long.

Culture characters: Culture was established from the germinating ascospore. Colony moderately growing on PDA media, reaching 2.5 cm after incubation for 16 days at 28 °C. The culture was sterile, white, entire, dense, raised, radially striated, cottony and reverse reddish brown.

Material examined: Thailand, Chiang Mai Province, Mushroom Research Center, on dead stem of an unidentified dicotyledonous climbing plant, 17 February 2019, Deping Wei, MRC13 (MFLU 21-0114, holotype); ex-type living culture MFLUCC 21-0111.

Notes: In the multigene phylogenetic tree, two strains of *F. cyperacearum* form a sister clade with significant support (99% MLBS/1.00 PP). Our new species, *Fitzroyomyces hyaloseptisporus* (MFLU 21-0114), forms a distinct clade nested between *F. cyperacearum* and *F. pandanicola* with middle support (71% MLBS/0.99 PP) (Figure 2). However, *F. hyaloseptisporus* differs from *F. cyperacearum* (MFLU 17-1480) in having small apothecia, different excipulum textura, septate paraphyses, larger asci and greater finely guttulate ascospores with more septations (Table 2). *Fitzroyomyces hyaloseptisporus* can be distinguished from *F. pandanicola* by its smaller apothecia, thinner excipulum with textura angularis, septate, wider paraphyses and smaller guttulate ascospore with more septations (Table 2). The sequence comparisons show 37 bp (4.3%) differences in a 844 bp fragment of LSU and 50 bp (6.5%) differences in a 765 fragment of ITS between *Fitzroyomyces hyaloseptisporus* and the type strain of *F. cyperacearum* (CBS 143170). The nucleotide differences between *Fitzroyomyces hyaloseptisporus* and *F. pandanicola* (HKAS 96206) were 32 bp (3.8%) in LSU (837 bp) and 33 bp (6.1%) in ITS (540 bp) regions. *Fitzroyomyces cyperacearum* is the only species that was represented by multiple strains (namely MFLU 18-0695b [37], MFLUCC 17-2072 [36] and CBS: 143170 [50]) that have low genetic diversity in the LSU region with 0.4% (5/1010 bp) difference and with 0.6% (4/595 bp) differences in the ITS region, suggesting that there is low intraspecific variation within *Fitzroyomyces*. Based on the guidance of Jeewon and Hyde [71], a minimum of >1.5% nucleotide differences in the ITS regions may be indicative of a new species. Therefore, the evidence from phylogenetic analyses, morphological observation and comparison of nucleotide sequences supports the establishment of a new species.

*Fitzroyomyces pandanicola* (Tibpromma and K.D. Hyde), D. P. Wei and K.D. Hyde, comb. nov.

Basionym: *Stictis pandanicola* Tibpromma and K.D. Hyde, 93: 78 (2018).

Index Fungorum number: IF 558679; Facesoffungi number: FoF 10096.

Notes: *Stictis pandanicola* was introduced by Tibpromma et al. [25] from a dead leaf of *Pandanus* sp. in Xishuangbanna, Yunnan Province, China. *Stictis pandanicola* formed a distinct clade restricted to the genus *Stictis* in their phylogenetic analyses [25]. *Stictis* is a polyphyletic genus whose natural classification remains unresolved [13]. *Stictis pandanicola* clustered with the extant species of *Fitzroyomyces* in this study and formed a distinct clade with maximum bootstrap support (100% MLBS/1.00 PP; Figure 2). *Stictis pandanicola* shares similar characteristics with *Fitzroyomyces* such as immersed and cupulate apothecia, a crystalliferous exciple, cylindric-clavate asci with thickened apices and cylindrical-filiform, multiseptated and hyaline ascospores. Thus, *S. pandanicola* is transferred to *Fitzroyomyces* based on phylogenetic and morphological evidence.

#### 3.2.3. *Ostropomyces* Thiyagaraja, Lücking, Ertz and K.D. Hyde, in Thiyagaraja, Lücking, Ertz, Karunarathna, Wanasinghe, Lumyong and Hyde, Journal of Fungi 7(no. 105): 11 (2021)

Index Fungorum number: IF 556555, two morphological species and two species with molecular data.

Type species: *Ostropomyces pruinosellus* Thiyagaraja, Lücking, Ertz and K.D. Hyde (2021).

Notes: *Ostropomyces* was introduced by Thiyagaraja et al. [20] based on *O. pruinosellus* and *O. thailandicus*. Both species were discovered on the same unidentified dead stem in Chiang Mai, Thailand. In this study, the phylogenetic analyses of LSU, ITS and mtSSU combined data (Figure 2) showed that the sexual isolates (MFLUCC 21-0112 and MFLU 21-0115) clustered with *O. pruinosellus*, while the asexual ones (MFLUCC 21-0113 and MFLU 21-0116) grouped with *O. thailandicus*. Both our asexual and sexual isolates were collected from an unidentified climbing plant in Chiang Rai, Thailand. These species are in close association with each other and appear during the dry season (February to March in northern Thailand). It is worth mentioning that the ascomata of *O. pruinosellus* somehow differed from general perithecioid ascoma morphology in developing an exposed disc, with the hymenium being entirely encompassed by excipulum. At first glance, this kind of ascoma resembles apothecioid ascoma. Here, we provide molecular and morphological data as well as culture characteristics for *O. pruinosellus* and *O. thailandicus*. All three strains of *O. pruinosellus* grouped together and branched seperately from *O. thailandicus* in the combined dataset phylogenetic tree (Figure 2). Three strains of *O. pruinosellus* differed by 0.8% (7/788 bp) in LSU and 4.3% (23/528 bp) in ITS. Three strains of *O. thailandicus* differed by 0.4% (4/833 bp) in LSU and 2.8% (17/590 bp) in ITS. Two strains (MFLUCC 21-0112 and MFLU 21-0115) of *O. pruinosellus* differed by 1.9% (14/726 bp) in mtSSU. Two strains (MFLUCC 21-0113 and MFLU 21-0116) of *O. thailandicus* were completely identical within 713 bp of mtSSU. *Ostropomyces pruinosellus* (MFLUCC 21-0112) and *O. thailandicus* (MFLUCC 21-0113) differed by 2.1% (18/840 bp) in LSU, 8.1% (46/567 bp) in ITS and 10% (77/706 bp) in mtSSU. The low level of intraspecific diversity and higher interspecific diversity strongly suggests that *O. pruinosellus* and *O. thailandicus* are not conspecific.

*Ostropomyces pruinosellus* Thiyagaraja, Lücking, Ertz and K.D. Hyde, in Thiyagaraja, Lücking, Ertz, Karunarathna, Wanasinghe, Lumyong and Hyde, Journal of Fungi 7(no. 105): 12 (2021) Figure 4.

Index Fungorum number: IF 556556; Facesoffungi number: FoF 10097.

Saprobic on the dead stem of an unidentified climbing plant. Sexual morph: *Ascomata* 230–300 × 300–365 μm ($\bar{x}$ = 265 × 330, *n* = 5), perithecial, subglobose, unilocular, gregarious, immersed at first and raising the substrates into small pustules and later opening by entire pores, exposing the discs. *Discs* pale grey to olivaceous, convex and covered by thick pruinose substance. Inner mass creamy-yellow. *Exciple* 25–50 μm ($\bar{x}$ = 40, *n* = 20), comprising interwoven and hyaline hyphae without crystalline inclusions and periphysoidal layers. *Hymenium* consisted of paraphyses and asci, lying parallel to the substrates. *Paraphyses* 1–3 μm ($\bar{x}$ = 2, *n* = 30) wide, anastomosing, aseptate, branched and longer than asci. *Asci* 180–265 × 9–15 μm ($\bar{x}$ = 220 × 12, n = 20), cylindrical, unitunicate and thick-walled, with a thickened cap. *Apical caps* 5–6.5 × 3–4.5 μm ($\bar{x}$ = 5.5 × 3.7, *n* = 5), hemispheric and pierced by a pore. *Ascospores* hyaline, filiform, as long as asci and easily disarticulating into part-spores in mature asci. *Part-spores* 2.5–6 × 2–3 μm ($\bar{x}$ = 4 × 2.5, *n* = 40), subglobose to ellipsoidal and unicellular, with two oil droplets. Asexual morph: Undetermined.

Culture characters: Culture was established from germinating part-spores. The colony was slowly grown on PDA media, reaching 3 cm after incubation for two months in 28 °C. It was sterile, yellowish white, nearly circular, dense, umbonate, surface folded, cottony and reverse creamy-yellow.

Material examined: Thailand, Chiang Rai Province, Doilan District, on dead stem of an unidentified dicotyledonous climbing plant, 27 March 2019, Deping Wei, CR2706 (MFLU 21-0115); living culture MFLUCC 21-0112.

Notes: Our isolate (MFLU 21-0115) resembles *Ostropomyces pruinosellus* (MFLU 20-0539) in having subglobose, white-pruinose, perithecioid ascomata, cylindrical asci and filamentous, anastomosing paraphyses and filiform ascospores that disarticulate into numerous ellipsoidal part-spores. Phylogenetic analyses supported close affinity of our isolates (MFLU 21-0115 and MFLUCC 21-0112) to *Ostropomyces pruinosellus* (MFLU 20-0538), and the three were grouped together with maximum statistical support. Here, we consider our isolate as a new collection of *Ostropomyces pruinosellus* and provide additional molecular and morphological data as well as culture characteristics for this species.

*Ostropomyces thailandicus* Thiyagaraja, Lücking, Ertz and K.D. Hyde, in Thiyagaraja, Lücking, Ertz, Karunarathna, Wanasinghe, Lumyong and Hyde, Journal of Fungi 7(no. 105): 13 (2021) Figure 5.

Index Fungorum number: IF 556557; Facesoffungi number: FoF 10098.

Saprobic on dead stem of an unidentified climbing plant. **Asexual morph:** *Conidiomata* 100–300 × 100–200 μm ($\bar{x}$ = 200 × 150, *n* = 10), pycnidial, gregarious, completely immersed to erumpent, unilocular, ostiolate, with irregularly branched and confluent basal parts, showing variously shapes ranging from globose and flask-shaped to cordiform in the vertical section. They contained dark inner content visible with black dots on the host surface. The host epidermis around the conidiomata is white. *Conidiomatal wall* 10–20 μm ($\bar{x}$ = 15, *n* = 25), consisted of hyaline and interwoven hyphae without crystalline inclusions. *Conidiophores* reduced to conidiogenous cells. *Conidiogenous cells* 2–6 × 1–3 μm ($\bar{x}$ = 4 × 2, *n* = 50), arising from inner layer of the cavity, sub-cylindrical to ampulliform, hyaline, smooth-walled cell and monoblastic. *Conidia* 7.5–13 × 1.5–3 μm ($\bar{x}$ = 10 × 2, *n* = 40), hyaline, ellipsoidal and unicellular, with minute oil droplets. They are percurrently proliferating and produced in unbranched chains.

Culture characteristics: Culture was established from the germinating conidia. Colony slowly growing on PDA media, attaining 2 cm after incubation for two months in 28 °C. The culture was sterile, yellowish white, nearly circular, dense, raised, surface folded, cottony and reverse creamy-yellow. It produced yellow-white and dispersive crystal substances in agar.

Material examined: Thailand, Chiang Rai Province, Doilan District, on the dead stem of an unidentified dicotyledonous climbing plant, 27 March 2019, Deping Wei, CR2708 (MFLU 21-0116); living culture MFLUCC 21-0113.

**Figure 4:**
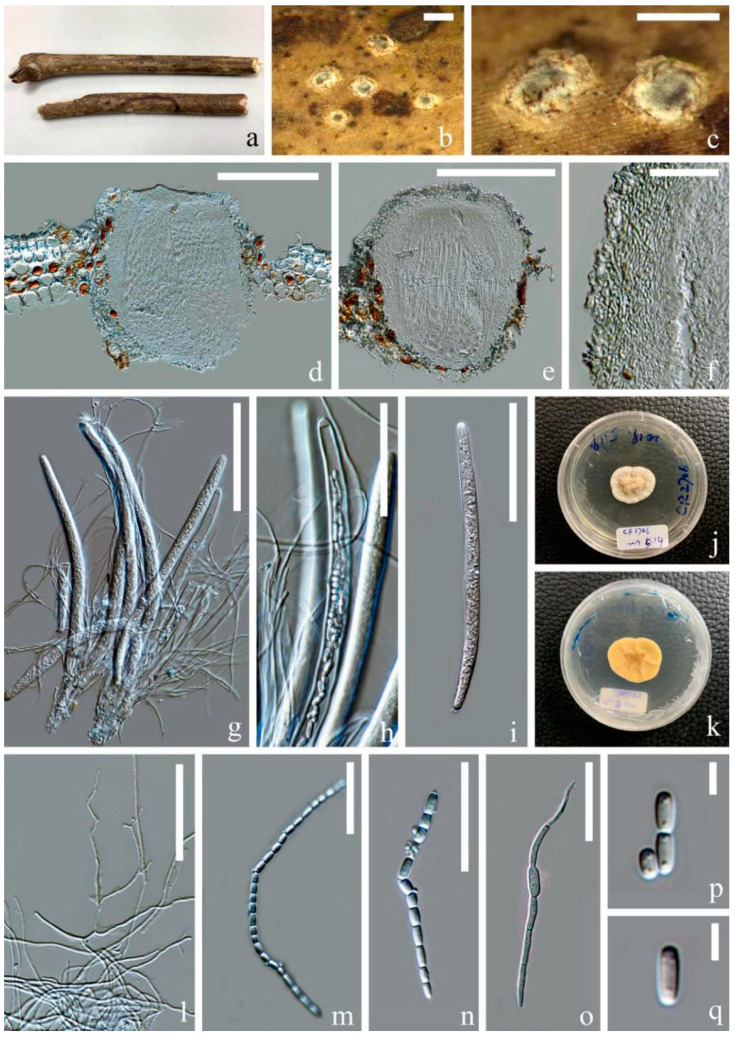
*Ostropomyces pruinosellus* (MFLU 21-0115). (**a**) Substrate; (**b**,**c**) ascomata on host; (**d**,**e**) vertical sections through ascomata; (**f**) excipulum; (**g**–**i**) Asci; (**j**,**k**) upper and lower view of culture on PDA media after incubation for two months; (**l**) paraphyses; (**m**,**n**) catenulate ascospores; (**o**) germinating ascospore; (**p**,**q**) part-spores. Scale bars: (**b**,**c**) = 500 μm; (**d**,**e**) = 200 μm; (**g**,**i**) = 100 μm; (**h**,**l**) = 50 μm; (**f**,**m**–**o**) = 30 μm; (**p**,**q**) = 5 μm.

Notes: In the ITS, LSU and mtSSU combined phylogenetic analysis, our strains MFLUCC 21-0113 and MFLU 21-0116 formed a clade with *Ostropomyces thailandicus* (MFLU 20-0539) with 100 MLBS/1.00 PP statistical support values (Figure 2). The MFLU 21-0116 isolate shares similar morphological characteristics to the type specimen (MFLU 20-0539) in having irregular-shaped conidiomata, hyaline, cylindrical conidiogenous cells and catenulate conidia that easily break into small, numerous and ellipsoidal units (Figure 4). Therefore, we introduced our isolate MFLU 21-0116 as a new collection of *O. thailandicus*. This species produces many crystal-like substances on PDA in vitro, which has rarely been reported in known Stictidaceae species. Additionally, based on the collections in this study and Thiyagaraja et al. [20], we found that *O. thailandicus* and *O. pruinosellus* likely occurred in close proximity to each other.

## 4. Discussion

It has been previously hypothesized that the common ancestor of Ostropales was lichenized and that the extant saprotrophic lineages are the result of multiple losses of lichenization [2,72]. Thiyagaraja et al. [20] also proposed that Stictidaceae was derived from loss of lichenization. In the phylogenetic analysis herein, we manually mapped lifestyles on the phylogenetic tree (Figure 2). Information on species lifestyles was acquired from relevant references [2,26]. Taxa included in the phylogenetic analysis herein comprised five lifestyles: (1) saprotrophic, (2) lichenized, (3) optionally lichenized, (4) lichenicolous and (5) endophytic. The present phylogenetic tree shows that saprobes are broadly dispersed throughout the tree; lichenized species disperse in six clades, optionally lichenized in three, lichenicolous in two and endophytic in one. The high diversity of life modes is notable even within genera; for example, within *Cryptodiscus* three lifestyles are present, while there are two within *Sphaeropezia* and *Stictis*. Collective consideration of previous findings and those herein supports the conclusions of Thiyagaraja et al. [20] regarding the saprobic nature of the common ancestor of Stictidaceae and the convergent development of non-saprobic life modes in this family. One, however, cannot draw firm conclusions at this point as 10 genera lack molecular data; thus, their phylogenetic position within Stictidaceae is undetermined. Notably some of these genera are parasitic, and it would be of interest to see their distribution across the tree. Regardless, the observed notable plasticity of lifestyles might represent a strategy of fungi adapted to habitats, where succession commonly occurs [55] and a driving force facilitating speciation in Stictidaceae [20].

In this study, we used sequences from 22 of the 32 genera in Stictidaceae to infer the phylogeny of this family (see Figure 2). Of these 22 genera, more than two taxa were sampled from *Cryptodiscus, Cyanodermella, Fitzroyomyces, Sphaeropezia*, *Schizoxylon* and *Stictis*, while this was not possible for the other 16 genera due to the lack of molecular data. The *Cryptodiscus* clade has maximum statistical support and comprises nine species, most of which have few-celled ascospores. These include *Cryptodiscus cladoniicola* (cylindrical to slightly fusiform, (2–)3(–4)-septate) [33], *C. epicladonia* (filiform to cylindrical, (5–)7–11-septate) [33], *C. foveolaris* (oblong, 1-septate) [40], *C. incolor* (cylindrical-clavate, 3−5-septate) [27], *C. gloeocapsa* (cylindrical-fusiform, 3–4-septate) [40], *C. muriformis* (ellipsoid, muriform) [28], *C. pallidus* (fusiform, 3-septate) [40], *C. pini* (oblong, 1-septate) [40] and *C. tabularum* (cylindrical to cylindric-fusiform, 3-septate)*. Cryptodiscus epicladonia and C. muriformis* are distinct within the genus in having filiform multiseptate ascospores and submuriform ascospores, respectively. The broad morphological diversity of the ascospore indicates that this character is not taxonomically significant for *Cryptodiscus.* The *Fitzroyomyces* clade contains three species, namely, *Fitzroyomyces cyperacearum, F. hyaloseptisporus* and *F. pandanicola.* All three species are similar in having long cylindrical ascospores with several prominent constrictions wherein the entire spore easily breaks into irregular fragments. The presence of these characters in all *Fitzroyomyces* species suggests that they might be useful diagnostic features of this genus. *Sphaeropezia* clade contains four species, including *Sphaeropezia leucocheila* (oblong-elliptic, 1-septate) [73], *S. lyckselensis* (cylindrical oblong, 3-septate) [42], *S. mycoblasti* (ellipsoidal, 3-septate) [42] and *S. shangrilaensis* (fusoid to obovoid, aseptate) [20]. The four species form short, nearly cylindrical and few-celled (0–3-septate) ascospores, indicating that the ascospore morphology is consistent and is, thus, a phylogenetically informative character within *Sphaeropezia*. Five *Stictis* species with filiform, multiseptated and non-disarticulating ascospores are included in our phylogenetic analyses forming at least four separate clades. Phylogenetic analyses in this and other studies suggest that *Stictis* should be subdivided into several genera [20]. Three *Schizoxylon* species were selected in the phylogenetic analysis herein. *Schizoxylon gilenstamii* and *S. berkeleyanum* formed a sister clade with high support, while *S. albescens* is sister to *Glomerobolus gelineus* with low support. In contrast to our findings, previous phylogenetic studies by Thiyagaraja et al. [20] and Fernández-Brime et al. [28] have recovered a monophyletic clade of *Schizoxylon*, albeit with weak support, implying that phylogenetic placement and membership of this genus remain unresolved. Additional studies and collections are necessary in order to figure out the phylogenetically informative characters at the species and genus level within Stictidaceae.

Of the ten genera that lack molecular data, *Conotremopsis, Delpontia* and *Propoliopsis* are monotypic. *Conotremopsis* is distinct in having superficial, elongated and keeve-like apothecia [34]. *Delpontia* has submuriform ascospores similar to those in *Cryptodiscus* and *Topelia.* Nonetheless, *Delpontia* can be easily distinguished from *Topelia.* The former forms wide-opening apothecia and has a non-lichenized lifestyle, while the latter has closed perithecioid apothecia and a lichenized lifestyle [13,43]. *Cryptodiscus* is diverse in terms of ascospore character morphology, apothecia nature and lifestyle [13,27,28], which can result in confusion as to its relationship with *Delpontia.* The other genera lacking molecular data are not monotypic. *Dendroseptoria* is distinct in its sporodochial fruiting bodies and staurosporous conidia [48], while *Stictophacidium* is set apart in having unicellular ascospores [13]. *Biostictis*, *Karstenia*, *Lillicoa* and *Nanostictis* have long clavate, cylindrical to filiform and multiseptate ascospores that resemble those of the majority of species in *Stictis* and *Schizoxylon* [13,35]. When taking shape and septation of ascospores into consideration, there is no clear-cut boundary among these eight genera. However, they can be distinguished based on other features. For instance, all members of *Biostictis* are reported as parasites of living leaves of plants [13]. *Karstenia* is unique in that it forms covering layers that consist of short-celled, vertically oriented hyphae ending in a fringe of hair-like projections [13]. All members of *Lillicoa* inhabit living leaves and seemingly are not lichenized. Conversely, all members of *Nanostictis* are obligately lichenicolous [13]. Hence, features such as lifestyle, substrate preference, nature of apothecia/conidiomata, structure of exciple and ascospore/conidia morphology are of taxonomic significance either independently or in combination for specific taxa. Molecular data are needed to examine the value of these diagnostic characters in intrageneric and intergeneric classification.

Sexual–asexual connections have been established for only a few genera of Stictidaceae including *Stictis* and *Acarosporina* using cultures and molecular evidence [28,52,53]. In *Schizoxylon pseudocyanosporum*, the asexual conidiomata were linked to its sexual apothecia solely on the basis of their close proximity to each other [13,53]. The connection, however, has not been confirmed by using pure cultures or molecular data. Similarly, this study and Thiyagaraja et al. [20] reported the intermingled occurrence of both sexual and asexual morphs together on the same substrate. Although it could be speculated that the two morphs belong to the same species, this is not very likely. Molecular data derived from pure cultures and subsequent sequence and phylogenetic analyses (see notes, Figure 2) collectively suggest that the two morphs are in fact separate species. In the phylogenetic analysis, the sexual morph grouped with *Ostropomyces pruinosellus*, while the asexual morph clustered with *O. thailandicus*. Moreover, the interspecific genetic diversity of all genetic markers used in this study between *O. pruinosellus* and *O. thailandicus* clearly indicates that the two are not conspecifics. Thus, establishing sexual-asexual links should not be based solely on the close proximity of the two morphs, as in the case of *S. pseudocyanosporum*, but supplemented with molecular data.

In this study, we have explored the taxonomic significance of characters for Stictidaceae. However, limited taxa sampling in the phylogenetic analyses hinders us from drawing a firm conclusion on this point. Additionally, taxonomic placements of pathogenic/parasitic species in Stictidaceae are as yet unclear due to the lack of molecular data. It would be interesting to observe if the addition of these taxa will confirm the saprobic nature of the ancestor of this family. *Stictis* contains many species, which were introduced solely on the basis of morphological observation, so gaps remain in terms of its natural classification using molecule-based phylogeny. Therefore, additional collections especially of early branching and under-sampled species are urgently needed in the future to address the issues mentioned above.

## Figures and Tables

**Figure 5:**
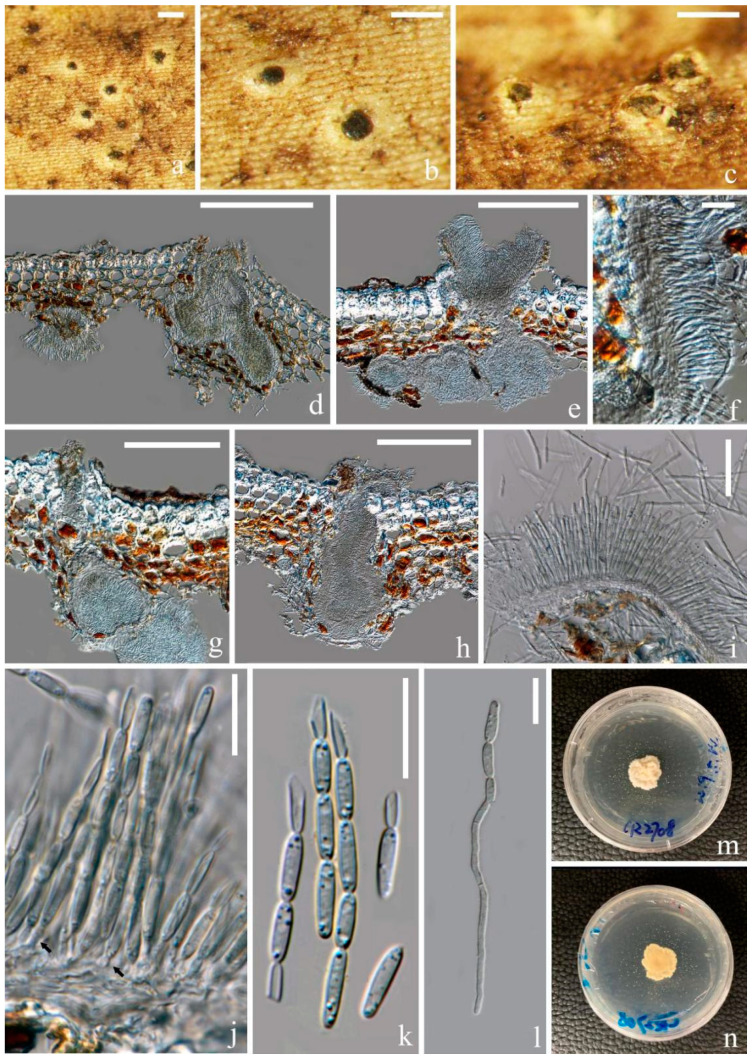
*Ostropomyces thailandicus* (MFLU 21-0116) (**a**–**c**) Conidiomata on host; (**d**,**e**,**g**,**h**) vertical sections through conidiomata; (**f**) conidiomatal wall; (**i**,**j**) conidiogenous cells (indicated with black arrows); (**k**) conidia; (**l**) germinal conidia; (**m**,**n**) upper and lower view of culture on PDA media after incubation for two months. Scale bars: (**a**–**d**) = 200 μm; (**e**,**g**,**h**) = 150 μm; (**i**) = 50 μm; (**f**,**j**–**l**) = 15 μm.

**Table 1:** GenBank accession numbers of the taxa used in the phylogenetic analyses in this study.

Species	Strain Number	LSU	ITS	mtSSU
*Absconditella sphagnorum*	M24	EU940095	-	EU940247
*Absconditella sphagnorum*	Palice 3820	AY300825	-	AY300873
*Acarosporina microspora*	AFTOL-ID 78	AY584643	DQ782834	AY584612
*Carestiella socia*	GG2410	-	AY661687	AY661677
*Carestiella socia*	GG2437a	-	AY661682	AY661678
*Carestiella socia*	Wedin 7194	-	-	JX266155
*Chromatochlamys muscorum*	Palice 8634	AY607731	-	AY607743
*Cryptodiscus cladoniicola*	RP160	KY661653	KY661620	KY661675
*Cryptodiscus cladoniicola*	RP159	KY661652	KY661619	KY661674
*Cryptodiscus epicladonia*	RP208 ^T^	-	KY661628	KY661680
*Cryptodiscus foveolaris*	EB155	-	FJ904673	FJ904695
*Cryptodiscus gloeocapsa*	EB93	-	FJ904674	FJ904696
*Cryptodiscus incolor*	EB164	-	FJ904675	FJ904697
*Cryptodiscus muriformis*	H.B. 6773	MG281963	MG281963	MG281973
*Cryptodiscus pallidus*	EB173	-	FJ904680	FJ904702
*Cryptodiscus pallidus*	EB152	-	FJ904679	FJ904701
*Cryptodiscus pini*	EB181	-	FJ904684	FJ904706
*Cryptodiscus pini*	EB178	-	FJ904683	FJ904705
*Cryptodiscus tabularum*	CO205	-	FJ904690	FJ904712
*Cryptodiscus tabularum*	EB169	-	FJ904689	FJ904711
*Cyanodermella asteris*	03HOR06-2-4 ^T^		KT758843	KT758843
*Cyanodermella banksiae*	CPC:32105 ^T^	NG_064548	NR_159835	-
*Cyanodermella oleoligni*	CBS 140345 ^T^	NG_058973	NR_153930	KX999144
*Cyanodermella viridula*	EB146	HM244763	-	HM244739
*Eriospora leucostoma*	CPC:35594	MT223890	MT223795	-
*Fitzroyomyces cyperacearum*	MFLU 18-0695b	MK499361	MK499349	-
*Fitzroyomyces cyperacearum*	CBS 143170 ^T^	NG_058513	NR_156387	-
***Fitzroyomyces hyaloseptisporus***	**MFLUCC 21-0111 ^T^**	**MZ868921**	**MZ868916**	**MZ868911**
*Stictis pandanicola*	HKAS 96206 ^T^	MH260319	MH275085	-
*Geisleria sychnogonoides*	Caceres and Aptroot 13560 (ABL)	KC689752	-	KC689751
*Geisleria sychnogonoides*	70627	KF220304	-	KF220306
*Glomerobolus gelineus*	JK 5584C	DQ247798	-	DQ247783
*Glomerobolus gelineus*	AFTOL-ID 1349	-	DQ247782	DQ247784
*Hormodochis aggregata*	CPC 30683^T^	MN317280	NR_166307	-
*Hormodochis melanochlora*	CBS 138861 ^T^	NG_070381	NR_165507	-
*Ingvariella bispora*	MALich 15288	HQ659184	-	HQ659173
*Ingvariella bispora*	BCNLich 17183	HQ659185	-	HQ659174
*Neofitzroyomyces nerii*	CBS 145088 ^T^	NG_068278	NR_161144	-
*Neostictis nigricans*	MFLU 18-1380 ^T^	MT214610	MT310654	-
*Orceolina kerguelensis*	Søchting 9398(C)	AY212830	AY212814	AY212853
*Ostropa barbara*	SW071 (S)	HM244773	HM244773	HM244752
*Ostropa barbara*	S F302817	MG281965	MG281965	MG281974
*Ostropomyces pruinosellus*	MFLU 20-0538 ^T^	MW400966	MW400964	
***Ostropomyces pruinosellus***	**MFLUCC 21-0112**	**MZ868917**	**MZ868912**	**MZ868907**
***Ostropomyces pruinosellus***	**MFLU 21-0115**	**MZ868918**	**MZ868913**	**MZ868908**
*Ostropomyces thailandicus*	MFLU 20-0539 ^T^	MW397060	MW400967	-
***Ostropomyces thailandicus***	**MFLUCC 21-0113**	**MZ868919**	**MZ868914**	**MZ868909**
***Ostropomyces thailandicus***	**MFLU 21-0116**	**MZ868920**	**MZ868915**	**MZ868910**
*Phacidiella alsophilae*	CPC:37041 ^T^	MT373344	MT373361	-
*Placopsis perrugosa*	KS137	KU844613	KU844737	KU844549
*Robergea cubicularis*	G.M. 2017-10-12.1	MN833317	MN833317	-
*Robergea cubicularis*	G.M. 2013-05-09.1	KY611899	KY611899	-
*Schizoxylon albescens*	GG2443a	AY661690	AY661690	AY661681
*Schizoxylon berkeleyanum*	S F209682	MG281966	MG281966	MG281975
*Schizoxylon gilenstamii*	S F300892	MG281968	MG281968	MG281977
*Sphaeropezia leucocheila*	PDD:98299 ^T^	MK547099	MK547090	MK547101
*Sphaeropezia lyckselensis*	EB-2012a	JX266158	-	JX266156
*Sphaeropezia mycoblasti*	EB-2012b	JX266159	-	JX266157
*Sphaeropezia shangrilaensis*	MFLU 20-0537 ^T^	MW400965	MW400955	MW400962
*Stictis anhuiensis*	HOU1233M	KX447623	-	KX447625
*Stictis anhuiensis*	HOU1233	KX447622	-	KX447624
*Stictis brunnescens*	GG2359	AY661688	AY661688	AY661679
*Stictis confusa*	Wedin 7070	-	NR_121318	DQ401141
*Stictis mollis*	MW7200	AY527313	AY527313	-
*Stictis mollis*	GG2445a	AY527318	AY527318	-
*Stictis populorum*	GG2610a	AY527327	AY527327	AY527356
*Stictis populorum*	GG2618 ^T^	AY527331	AY527331	AY527360
*Stictis radiata*	GG2449a	AY527308	AY527308	-
*Stictis radiata*	MW6493	AY527309	AY527309	-
*Stictis urceolatum*	LT21500	AY661686	AY661686	AY661676
*Thelenella antarctica*	Lumbsch 19006a (F)	AY607739	-	AY607749
*Trapelia placodioides*	KS163	KU844623	KU844758	KU844568
*Trinathotrema stictideum*	F:Luecking 17541b	-	-	GU380288
*Trinathotrema stictideum*	F:Luecking 28093	-	-	GU380287
*Xyloschistes platytropa*	AFTOL-ID 4891	KJ766680	-	KJ766517

The newly generated sequences are in bold font. The type strains are indicated with the symbol “T”.

**Table 2:** Morphological comparison of *Fitzroyomyces* species.

Species	Strain	Apothecia (μm)	Exciple (μm)	Paraphyses (μm)	Asci (μm)	Ascospores (μm)	Septation	Reference
*F. cyperacearum*	MFLU 17–1480	201–260 × 210–310	17–70, textura intricata	1.3–3, aseptate	110–150 × 10–20	100–145 × 2.5–3.5, eguttulate	17–21	[36]
*F. hyaloseptisporus*	MFLU 21–0114	140–200 × 150–200	6–20, textura angularis	1–3, septate	165–200 × 10–25	150–200 × 3.5–6, finely guttulate	up to 58	this study
*F. pandanicola*	HKAS 96206	350–410 × 520–650	27–46, textura epidermoidea	0.8–1.1, aseptate	160–240 × 7.5–23	190–265 × 4–5, eguttulate	up to 40	[25]

## Data Availability

Not applicable.
